# Energetic Benefits of Sociality Offset the Costs of Parasitism in a Cooperative Mammal

**DOI:** 10.1371/journal.pone.0057969

**Published:** 2013-02-25

**Authors:** Heike Lutermann, Nigel C. Bennett, John R. Speakman, Michael Scantlebury

**Affiliations:** 1 Mammal Research Institute, Department of Zoology and Entomology, University of Pretoria, Hatfield, South Africa; 2 Institute of Biological and Environmental Sciences, University of Aberdeen, Zoology Building, Aberdeen, United Kingdom; 3 School of Biological Sciences, Queen's University Belfast, Belfast, United Kingdom; 4 State Key Laboratory of Molecular and Developmental Biology, Institute of Genetics and Developmental Biology, Chinese Academy of Sciences, Beijing, People's Republic of China; CNRS, Université de Bourgogne, France

## Abstract

Sociality and particularly advanced forms of sociality such as cooperative breeding (living in permanent groups with reproductive division of labour) is relatively rare among vertebrates. A suggested constraint on the evolution of sociality is the elevated transmission rate of parasites between group members. Despite such apparent costs, sociality has evolved independently in a number of vertebrate taxa including humans. However, how the costs of parasitism are overcome in such cases remains uncertain. We evaluated the potential role of parasites in the evolution of sociality in a member of the African mole-rats, the only mammal family that exhibits the entire range of social systems from solitary to eusocial. Here we show that resting metabolic rates decrease whilst daily energy expenditure and energy stores (i.e. body fat) increase with group size in social Natal mole rats (*Cryptomys hottentotus natalensis*). Critically, larger groups also had reduced parasite abundance and infested individuals only showed measurable increases in energy metabolism at high parasite abundance. Thus, in some circumstances, sociality appears to provide energetic benefits that may be diverted into parasite defence. This mechanism is likely to be self-reinforcing and an important factor in the evolution of sociality.

## Introduction

The energy costs of parasitism are presumed to arise as a result of the physiological consequences of mounting an immune response, the costs of repairing tissue damage and/or direct resource competition with the host [1]–[4]. Thus, resting metabolic rate (RMR) and daily energy expenditure (DEE) are often [5], [6], but not always [7]–[10], higher in animals that are parasitized compared with those that are not. It is generally assumed that organisms living in larger social groups suffer greater levels of parasitism and infection than solitary ones due to the density-dependent nature of parasite transmission [11]–[15]. Indeed, comparative studies suggest that parasite burden (i.e. diversity, prevalence and/or abundance) increases with group size [14]–[17]. As a consequence, the increased risk of parasitism and associated energetic costs may present an important constraint on the evolution of group-living or sociality [12], [18], [19].

Group-living entails a number of individuals that live and/or interact with each other. However, the duration and characteristics of groups can vary widely and encompass the large temporary breeding aggregations found in many seabirds as well as species that live in permanent groups with well established social relationships [19]. Only the latter groups are considered truly social for the purpose of our study. Sociality reaches its pinnacle in cooperatively breeding species where groups are composed of closely related members and is characterized by a reproductive division of labour, overlapping generations and cooperative care for young [20]. The benefits of sociality, such as increased predator detection, offspring survival, foraging efficiency and social thermoregulation are well documented in vertebrates [21]–[24] and most of these benefits are intimately linked to an individual's energy budget. Therefore, sociality may evolve if the energetic benefits of group-living offset the energetic costs of elevated levels of parasitism. Indeed, if the energetic benefits of sociality are large, animals may be able to channel additional resources into combating parasite impact and to minimize parasite burden. However, as far as we are aware no study has yet quantified the energetic benefits of sociality and compared them with the potential costs of parasitism. Here we set out to investigate both sides of this balance in a cooperatively breeding rodent as a model species.

Natal mole-rats (*Cryptomys hottentotus natalensis*) are subterranean rodents that live in colonies of 2–15 and breed year-round [25]. They exhibit a high reproductive skew and only one female and up to three males per colony may breed [26]. Evidence suggests that the subterranean niche strongly limits the number of parasite species a host species is exposed to [27]–[29] suggesting that a social, subterranean rodent may be an ideal model to test for effects of sociality on parasite burdens and energy budget. Indeed, only two species of gastrointestinal endoparasites and one genus of ectoparasite have been found in Natal mole-rats. Cestodes of the genus *Raillietina* were the most common parasites while prevalence and abundance of other parasite species were low (see Materials and Methods) suggesting that parasites other than *Raillietina* sp. are unlikely to exert a great impact on the energy budget of Natal mole-rats. We tested the suggestion that sociality generates energetic benefits which offset the cost of parasitism by measuring the resting metabolic rate (RMR) and cestode abundance (i.e. the mean number of parasites per individual) of wild Natal mole-rats of different colony sizes. We also measured daily energy expenditure (DEE) as well as body fat content in all individuals. We predicted that larger colony sizes would be associated with higher parasite abundance due to higher transmission rates. Further, we predicted that infection with *Raillietina* sp. would result in increased RMR's and DEE's and decreased fat stores, and that such effects would be more pronounced in individuals experiencing some form of constraint on energy allocation (e.g. during winter or whilst breeding). We also hypothesized that larger colony sizes would be associated with energetic benefits. If these benefits reduce metabolic costs then we predicted that DEE would decrease with colony size whilst fat stores would be increased in larger colonies. This then might offset the elevated costs of parasitism leading to no overall relationship between group size and daily energy demands.

## Results

### (a) Parasite burden

**Figure 1:**
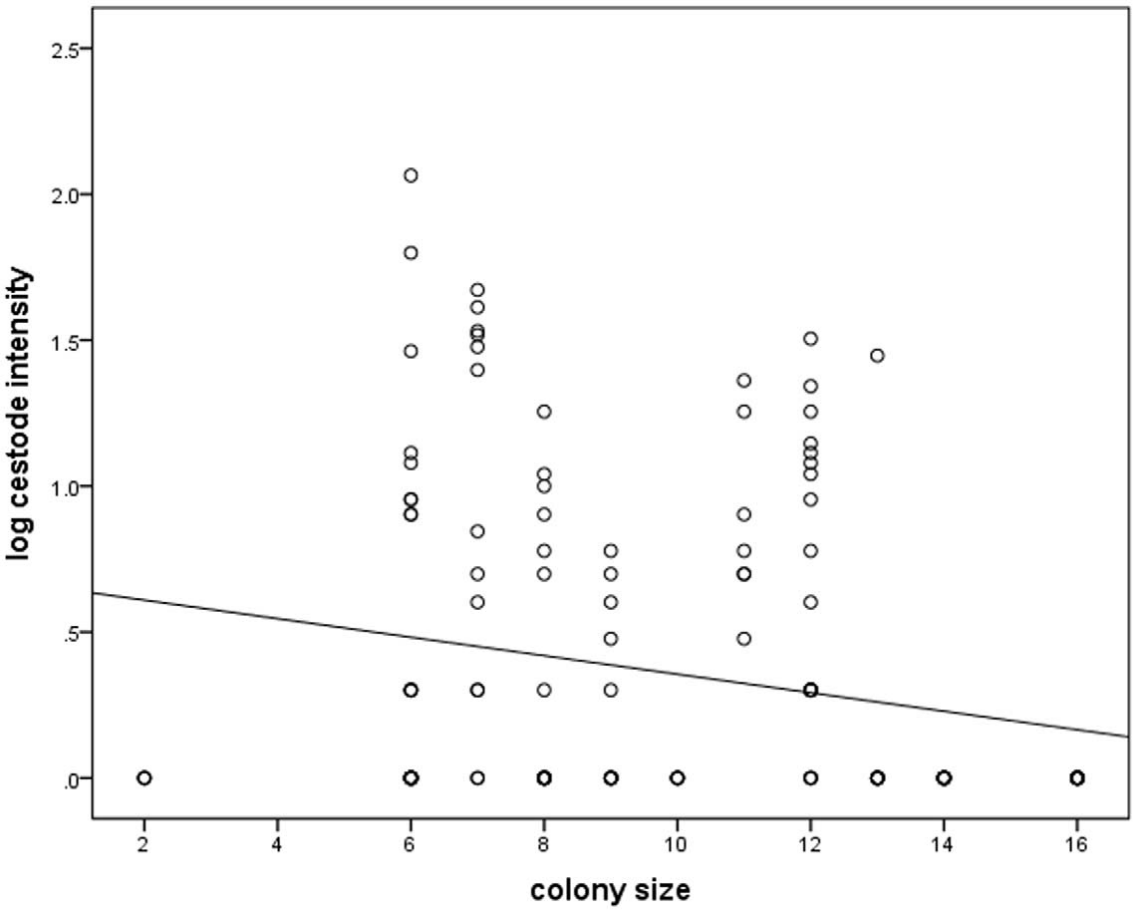
Correlation between Log_10_ parasite abundance and colony size for 148 individuals from 25 colonies of known size (R_S_ = −0.263, p = 0.002).

Contrary to the general expectation, infection with *Raillietina* sp. was negatively associated with group size (Wald χ^2^ = 4.469, df = 1, p = 0.035, Fig. 1). There was a significant interaction between sex and breeding status with breeding females tending to have lower parasite abundance than breeding males (p = 0.055). Parasite abundance was also significantly higher in 2003 than 2006 (Wald χ^2^ = 6.682, df = 1, p = 0.010) and greater in winter compared to summer (Wald χ^2^ = 6.641, df = 1, p = 0.010).

### (b) Energy use and storage

**Figure 2:**
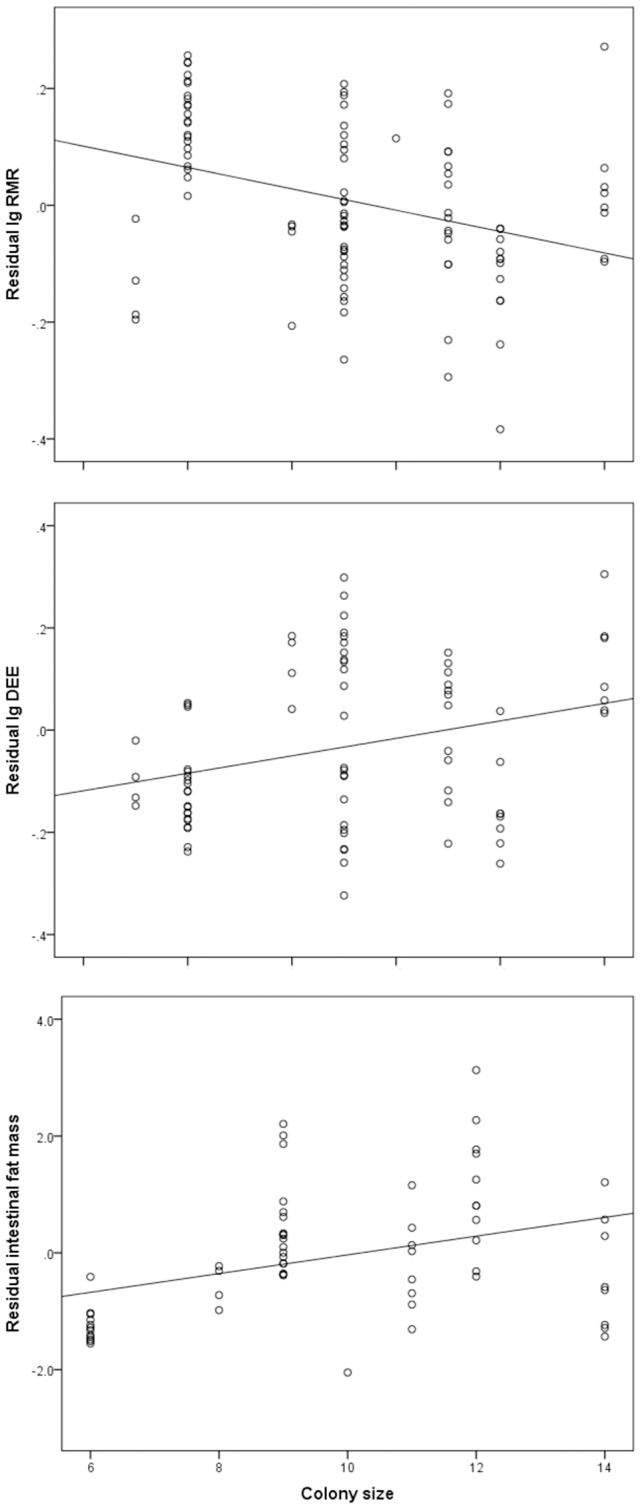
Correlations between group size and energetic measures. (a) Resting metabolic rate against colony size (residual log_10_RMR, kJ/day); (b) Daily energy expenditure against colony size (residual log_10_DEE, kJ/day); and (c) Fat mass against colony size (residual fat mass, g) of *C. h. natalensis*.

**Figure 3:**
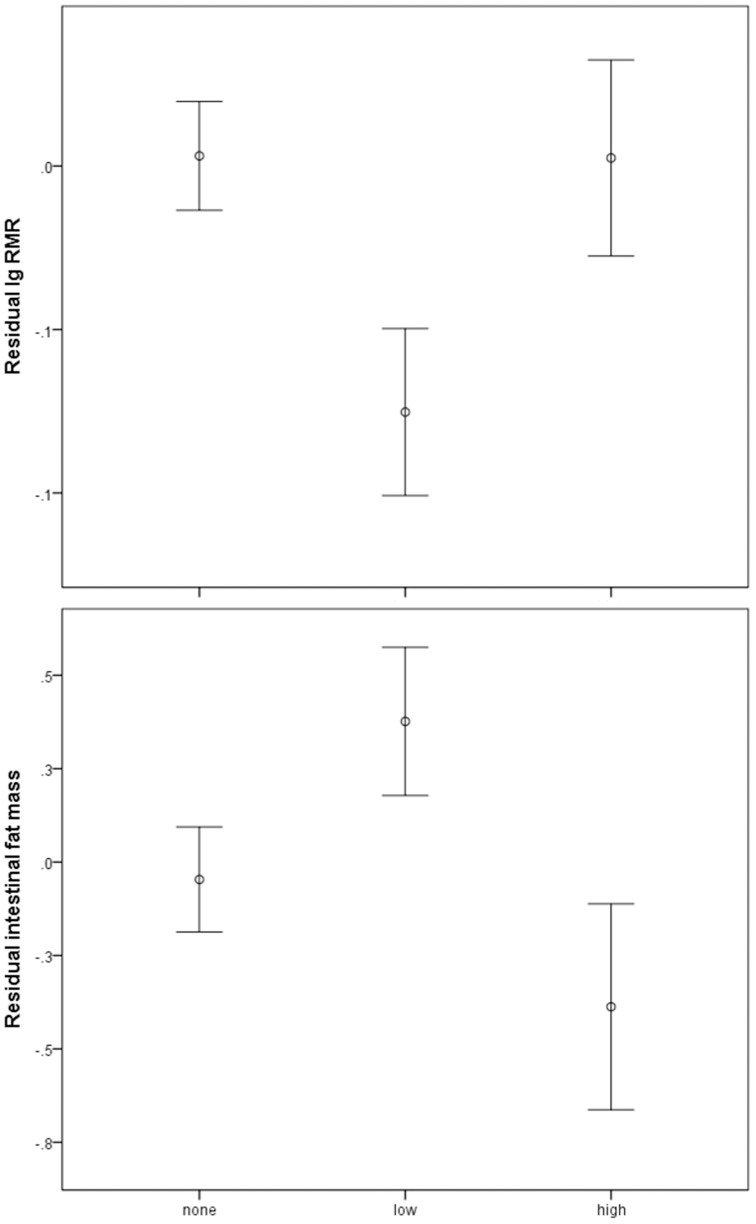
Variation in (a) resting metabolic rate (residual log_10_RMR, kJ/day) and (b) fat mass (residual fat mass, g) with cestode abundance in *C. h. natalensis*. Displayed are means ± SD.

Resting metabolic rate decreased with increasing colony size (F_1,10_ = 46.50, p<0.0001, Fig. 2a). In addition, RMR was significantly affected by parasite abundance (F_2,43_ = 13.00, p<0.0001) and RMR was lower for animals with low cestode abundance than both individuals that were not infested and those that sustained high cestode abundance (Fig. 3a). Breeders had significantly higher RMR values than non-breeders (F_1,45_ = 55.57, p<0.0001). There was a significant positive correlation between RMR and body mass (F_1,45_ = 6.71, p = 0.013; least squares regression: RMR (kJ/day)  = 0.746+0.534× body mass (g), r^2^ = 0.20). RMR values were 10.2% lower than allometric predictions for asmall mammal of similar size [30]. Once the effects of mass had been accounted for, there was no difference in RMR between breeders and non-breeders (t = −0.385, p = 0.701). RMR was greater in winter compared to summer (F_1,10_ = 10.05, p = 0.010) and there was a significant interaction between year and season (F_1,10_ = 26.12, p = 0.0005), indicating that RMR differed between years. None of the remaining factors affected RMR (Table 1).

**Table 1:** Minimal linear mixed models for the three energetic measurements.

	RMR		DEE		Fat mass	
	F	P	F	p	F	p
**Colony size^a^**	**46.50**	<0.0001*	**5.40**	**0.0452**	**12.19**	0.0058*
Year	4.51	0.0596	210.12	<0.0001*	0.14	0.7120
Season	10.05	0.0100*	-	-	12.34	0.0056*
Sex	-	-	6.64	0.0141*	-	-
Breeding Status	55.57	<0.0001*	13.15	0.0009*	-	-
Body mass	6.71	0.0130*	7.67	0.0215*	6.90	0.0118*
**Cestode abundance**	**13.00**	<0.0001*	-	-	**4.78**	0.0132*
Year*season	26.12	0.0005*	-	-	31.01	0.0002*
Year*sex	-	-	6.51	0.0150*	-	-

Effects of colony size, abundance, year, season, sex, breeding status and body mass on resting metabolic rate (RMR), daily energy expenditure (DEE) and fat mass. Suffix ‘a’ indicates colony size was included as a covariate, ‘–’ indicates that the variable was removed from the final model and ‘*’ indicates a significant effect.

The DEE increased significantly with colony size (F_1,9_ = 5.40, p = 0.045, Fig. 2b). In contrast, cestode abundance did not significantly affect DEE and was consequently dropped from the final model (Table 1). Females had significantly lower DEEs than males (F_1,37_ = 6.64, p = 0.014) and non-breeders had significantly lower DEEs than breeders (F_1,37_ = 13.15, p = 0.0009). There was a significant positive correlation between DEE and body mass [F_1,37_ = 7.67, p = 0.009; least squares regression: DEE (kJ/day)  = 0.990+0.572× body mass (g), r^2^ = 0.16]. However, once the effects of body mass had been accounted for, there was no difference in DEE between the sexes (t = −1.466, p = 0.147) or between breeders and non-breeders (t = −0.565, p = 0.574). The interaction between year and sex was significant (F_1,9_ = 6.51, p = 0.0150) indicating that DEE values differed between years. None of the remaining factors contributed significantly to variation in DEE (Table 1).

Fat mass was positively correlated with body mass (F_1,46_ = 6.77, p = 0.012) and decreased significantly with RMR (R = −0.548, n = 59, p<0.0001). However, there was no significant correlation between fat mass and DEE (R = 0.090, n = 50, p = 0.526). Fat mass increased significantly with colony size (F_1,10_ = 12.19, p = 0.006, Fig. 2c) and varied significantly with abundance (F_2,44_ = 4.78, p = 0.013). It was greater for animals with low cestode abundance than unparasitised individuals and those in the high cestode abundance category (Fig. 3b). Fat mass was significantly lower in winter than summer (F_1,10_ = 12.34, p = 0.006). There was a significant interaction between year and season (F_1,10_ = 31.01, p = 0.0002) indicating that fat mass differed between years.

## Discussion

Contrary to our *a priori* predictions, we found that mole-rats in larger colonies exhibited a lower abundance of *Raillietina* sp. This supports the hypothesis that larger groups may accrue benefits that either prevent transmission of this parasite or allow for more successful defence against it. Similar negative relationships between group size and parasite burden have been shown for directly transmitted ectoparasites and this has been attributed to increased allo-grooming rates with group size [27], [31]. In contrast, cestodes cannot be directly transmitted between hosts but require an intermediate host [32] and hence, the link between group size and parasite transmission is not as intuitive. However, it has been suggested that host aggregation around key resources may play a crucial role in the transmission of parasites with an indirect life-cycle [33], [34]. Due to their subterranean life-style the movements of mole-rats are largely limited to the shared burrow system. The nest, food chambers and latrines are likely focal points of activity [22] were encounters with the intermediate arthropod host of *Raillietina* sp. would be increased resulting in greater transmission rates in larger groups. Behavioural mechanisms such as those proposed for ectoparasites are unlikely to be efficient countermeasures reducing transmission rates in this scenario. Consequently, a more efficient physiological parasite defence in larger groups appears to be more likely. Such a mechanism can be particularly beneficial for social organisms as in the natural environment individuals are generally faced with challenges from more than one parasite species [35]. Infestation with one parasite can put substantial constraints on the ability of a host to respond to secondary infestations which are likely in the wild due to the ubiquity of more than one parasite species present in natural environments [36]. Similar effects of multiple parasite infestation have also been observed in a closely related mole-rat species, the highveld mole-rat (*Cryptomys hottentotus pretoriae*) [37]. Hence, energy savings obtained in larger groups may have played an important role in overcoming constraints to the evolution of sociality in bathyergids. Experimental manipulation of parasite burdens and/or group sizes could help to corroborate or refute this hypothesis.

Across mammalian species, there is a positive correlation between parasite species richness and basal metabolic rate and possibly because an increased basal immune investment could be beneficial when facing the potential challenge by a large and diverse number of parasite species [38]. While the low RMR and parasite species richness observed in the current study appears to concur with this pattern, the effects of parasitism on RMR were complex with the lowest RMR values observed in individuals with intermediate parasite loads. Unlike previous observations, we did not find a gradual increase in either RMR or DEE or a decrease in fat mass with increasing cestode abundance, although differences were apparent between individuals with low and high cestode abundance. Since parasite infections are thought to induce energetically expensive immune responses in their hosts [2], [3], this was unexpected. However, such immune responses are usually triggered during the initial parasite invasion and it is during this acute infestation when most experimental studies attempt to measure such costs. As the cestodes in our study were fully matured in the naturally infected study animals, they had presumably reached the chronic phase when hosts usually have already made physiological adjustments to maintain homeostasis [39], [40]. Moreover, it is well established that mature helminth parasites can down-regulate their host's immune system [41], [42] which in this case may account for the reduction in RMR in hosts with low cestode abundance. Gastrointestinal parasites may also induce anorexia [4], compete directly with their hosts for incoming food [43] and impair the efficiency of food absorption of the gastrointestinal tract [44]–[46] all of which may contribute to the observed reductions in RMR. Nevertheless, at high abundance tissue damage caused at attachment sites and the intense food competition (in our case, in some individuals the entire small intestine lumen was taken up by cestodes) may result in increased RMRs and reduced fat deposits compared to those of hosts with low cestode abundance. If animals adjust their activity budgets when infested [47], [48] this might account for a lack of variance of DEE with parasite abundance.

Our results show that the energetic implications of sociality can be significant, as all the measured variables were affected by colony size. As expected, there was a lower RMR and fat mass greater in mole-rats from larger groups. This potentially reflects the lower demands placed on the animals by parasite defence but also energetic benefits accrued from increased foraging efficiency that has previously been shown for cooperatively breeding mole-rats [22], [49]. This lowered RMR, however, did not translate into lowered daily energy demands. Indeed mole-rats from the larger colonies actually had higher daily energy expenditures. Variations in daily energy expenditure have traditionally been viewed in two contrasting ways [50]. On the one hand, larger expenditures may reflect the harshness of living; on the other hand, high expenditures may be enabled by lower constraints on expenditure – which could include resource supply from the environment [50] or fewer constraints on the capacity to dissipate heat [51]. In the present study we favour the second interpretation because not only did the animals from larger colonies have greater energy expenditures but they were also fatter – pointing to elevated resource availability for individuals living in the larger groups. The greater total DEE may then reflect greater physical activity levels associated with the energetic investment in subterranean foraging [52], [53] in the animals from larger colonies as they collected such resources.

We suggest that sociality in these mole-rats entails significant energetic benefits, some of which can be channelled into defence against parasites resulting in a negative relationship between parasite abundance and group size. Energetic efficiencies associated with sociality may therefore have been an important factor favouring the evolution of sociality in African mole-rats that comprise of only four solitary but more than 40 cooperatively breeding species [54]. Hence, the rarity of sociality in vertebrates in general might be due in part to the increased energetic costs of parasitism. Where social groups have evolved, some aspect of their group-living may have resulted in energetic benefits that have overcome these costs.

## Materials and Methods

### (a) Study site and Animals

The study area consisted of a 40 ha golf course surrounded by montane grassland at approximately 1500 m altitude in the Drakensberg mountain range, 64 km west of Mooi Rivier, KwaZulu-Natal South Africa (25°58'S; 21°49'E). Mole-rat tunnels were located underneath and adjacent to fresh molehills. Mole-rats were captured using Hickman live-traps [55] every two months from February 2003 to January 2004 and in March and July 2006. It took approximately four days to capture all individuals from a single colony and only colonies that contained both male and female breeders were considered complete [54]. Traps were left open for at least two days after the last animal had been captured to ensure that no more individuals remained underground. We cannot discount the possibility that this method resulted in an underestimation of group sizes. However, in our experience even one animal will block many tunnel entrances and create new mounds, especially when the tunnel system is disturbed. Since tunnel-blocking never occurred when all members of a colony were deemed to have been captured, we are confident that we did indeed capture all colony members. Animals were maintained in their colonies in plastic crates (80×40 cm wide ×50 cm high) with sawdust provided as bedding. Only adult individuals (body mass ≥40 g) were considered for analyses. Group size ranged from 4 to 14 and average colony size was 9.1±2.8. Cages were shaded from direct sunlight but were otherwise exposed to ambient conditions. Breeding females were readily distinguished by the presence of extended nipples, a perforate vagina and/or signs of pregnancy. Males were classified as breeders and non-breeders based on [26]. On completion of the metabolic measurements undertaken in the field, animals were taken back to the University of Pretoria for the assessment of parasite abundance and fat mass. Each individual contributed a maximum of one measurement to each of the variables indicated below. Experimental procedures and animal husbandry practices have been approved by the Animal Ethics Committee, University of Pretoria (AUCC 030110-002). Permission for animal capture was granted by KwaZulu-Natal Nature Conservation Services.

### (b) Resting metabolic rate (RMR)

All metabolic measures were carried out in the field. We used an open circuit respirometry system in which a chamber (1610 cm^3^) was immersed in a temperature-controlled water bath and maintained at 28–29°C (within the thermoneutral zone) [56]. Dried air was pumped into the chamber at 500 ml/min, controlled by an upstream flow regulator. Oxygen concentration was measured by an oxygen analyzer (S-2A Applied Electrochemistry). We determined RMR as minimal oxygen consumption when animals were seen to be at rest, after an initial hour in which they were familiarized to the chamber. These measurements were taken with three days of the initial capture of an individual. After RMR was determined, animals were injected with doubly labeled water (DLW) and released at their capture sites until recaptured for daily energy expenditure (DEE) determination (below). A total of 122 animals from 28 colonies were captured for initial measurements (i.e. body mass and RMR) of which 17 colonies were complete (n = 97 animals).

### (c) Daily energy expenditure (DEE)

We used the DLW technique [57], [58] to measure the DEE (kJ/day) of Natal mole-rats. Briefly, animals were blood sampled, weighed, and then injected IP with a known mass of DLW [100 g 95% APE enriched ^18^O water (Rotem Industries Ltd, Beer Sheva, Israel) and 50 g 99.9% APE enriched ^2^H water (Isotec Inc. Miamisburg OH, USA) mixed with 342 g ^1^H_2_^16^O; 0.3 g/100g]. Blood samples were taken again after 1 h to estimate initial enrichments. Animals were then released at the site of capture. After 48 h, traps were set and animals were recaptured over the next 5 days. Final blood samples were taken after whole 24-h periods to estimate isotope elimination rates, prior to calculation of DEE [59]. DEE measurements were obtained for 102 individuals from 23 colonies.

### (d) Fat mass and parasite abundance

After determination of DEE, animals were taken to the Department of Zoology, University of Pretoria where they were euthanized under terminal anaesthesia with halothane within less than two weeks of capture. The alimentary tracts of each individual were then removed and stored in 70% ethanol. Measures of fat mass were obtained as described in [26]. For parasite assessment, the alimentary tract was opened by lateral incision and the contents flushed out with 0.9% saline. The parasites were counted and stored in 70% ethanol. Parasite abundance was determined by dissection of the intestine for a total of 230 individuals from 59 colonies, of which 25 colonies were complete (148 animals). Identification was carried out by the Royal Veterinary College, London. Only two endoparasite species, the cestode *Raillietina* sp. (41.2%) and the nematode *Ascarops africana* (5.7%) were found in all animals dissected. As common for parasite data, cestode distribution were highly aggregated among hosts (mean ± SD: 3.31±8.37, range: 0–62). Hence, we grouped them in three categories (none: 0, few: 1–5, high: 6–62) for the analyses of cestode abundance on our energetic measures (see below).

### (e) Statistical analyses

Analyses were restricted to complete colonies as group size was only known for these individuals. Factors affecting variation in parasite abundance were assessed by fitting a generalized linear mixed model (GLMM) with a Poisson distribution and log- link function. Colony identity was included as random factor with colony size, year, season, sex and breeding status included as fixed factors. Body mass was entered as a covariate. The fit of the full model (AIC  = 927.63) was not improved by dropping non-significant variables (Akaike information criterion (AIC) [60] (minimal model: 927.17) and we therefore report the results for the former. We chose to fit a GLMM with a Poisson distribution rather than a GLM with a negative binomial distribution as data from the closely related highveld mole-rats suggest a strong effect of colony membership in parasite burden [27].

Possible effects of fat mass on the energetic measurements were assessed by carrying out partial correlations whilst controlling for body mass. For all statistical models, RMR and DEE were log_10_-transformed to meet the criteria for a normal distribution. We used linear mixed models (LMM) to examine differences in RMR, DEE and fat mass with colony identity as random factor to account for repeated measurements of several individuals from the same colony. Colony size and body mass were included as covariates in the models whilst year, season, reproductive status (breeder and non-breeder) and sex were included as categorical factors. All two-way interaction terms were initially included in the model. Terms were dropped sequentially using the AIC to achieve the model with the best fit [60]. In addition, abundance class was included in the models but no interactions were included to avoid parameter overload. Parameters were sequentially dropped from models using the AIC, [61] to obtain the minimal model and only the latter is reported. Post-hoc comparisons for significant variables were carried out employing t-tests.
